# Efficient and Security Enhanced Anonymous Authentication with Key Agreement Scheme in Wireless Sensor Networks

**DOI:** 10.3390/s17030644

**Published:** 2017-03-21

**Authors:** Jaewook Jung, Jongho Moon, Donghoon Lee, Dongho Won

**Affiliations:** Department of Computer Engineering, Sungkyunkwan University, 2066 Seoburo, Suwon, Gyeonggido 440-746, Korea; jwjung@security.re.kr (J.J.); jhmoon@security.re.kr (J.M.); dhlee@security.re.kr (D.L.)

**Keywords:** wireless sensor networks, session key agreement, off-line password guessing attack, lightweight computation, formal proof

## Abstract

At present, users can utilize an authenticated key agreement protocol in a Wireless Sensor Network (WSN) to securely obtain desired information, and numerous studies have investigated authentication techniques to construct efficient, robust WSNs. Chang et al. recently presented an authenticated key agreement mechanism for WSNs and claimed that their authentication mechanism can both prevent various types of attacks, as well as preserve security properties. However, we have discovered that Chang et al’s method possesses some security weaknesses. First, their mechanism cannot guarantee protection against a password guessing attack, user impersonation attack or session key compromise. Second, the mechanism results in a high load on the gateway node because the gateway node should always maintain the verifier tables. Third, there is no session key verification process in the authentication phase. To this end, we describe how the previously-stated weaknesses occur and propose a security-enhanced version for WSNs. We present a detailed analysis of the security and performance of our authenticated key agreement mechanism, which not only enhances security compared to that of related schemes, but also takes efficiency into consideration.

## 1. Introduction

**Figure 1:**
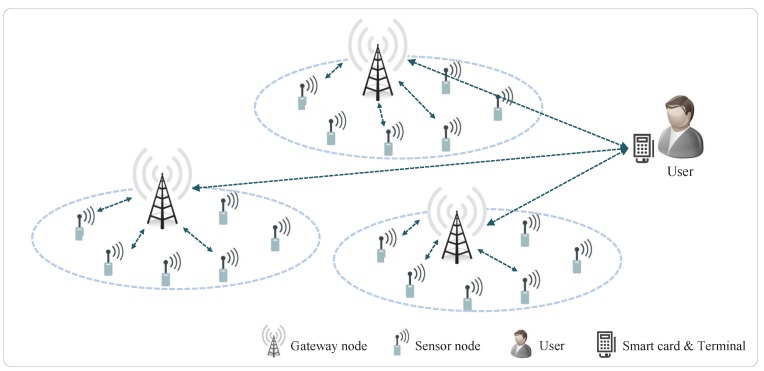
WSN system architecture.

Wireless Sensor Networks (WSNs) are distributed networks composed of tiny autonomous sensors capable of collecting information related to the environment or physical conditions of a target region [1]. WSNs can be implemented in various use cases—including military battlefields, healthcare services and smart grid networks—to provide convenience to users [2]. Figure 1 illustrates the WSN system architecture. As shown in Figure 1, WSN systems are comprised of three parties, including the user, the gateway nodes and the sensor nodes [1,2]. WSN is made of sensor nodes that are wirelessly connected to a gateway that is then connected to a user. On the other hand, in some WSNs, the sensor nodes can also be connected to each other in order to facilitate multi-hop wireless mesh networks.

Although users enjoy the simplicity and efficiency in WSNs, security has emerged as a major issue in both academia and industry [3]. Specifically, confidential information including the user’s identity and password should not be exposed even if an unauthorized user eavesdrops on data packets transmitted in the WSN [4]. To guarantee reliability among the communicating parties, an authentication mechanism can afford confidentiality and integrity when users access WSNs [3,4]. At this point, in order to design a secure authentication mechanism for WSNs, the following security requirements should be commonly considered [5,6,7,8,9,10,11,12,13].

- User anonymity: Even if an attacker extracts some information stored in the user’s smart card or if it eavesdrops on the messages transmitted in the communication group, the user’s identity should be protected.
- Mutual authentication: An authentication mechanism should execute several steps to achieve mutual authentication, which is to test all transmitted messages to judging the legitimacies.
- Session key agreement: After the mutual authentication process has completed, the session key should be securely assigned to communication parties on the network.
- Password verification process: If a user mistakenly enters an incorrect password in the login phase, the password should be promptly detected before performing the authentication phase.
- User friendliness: An authentication mechanism provides a password change procedure with which a user can freely update their password without communicating with the gateway node.
- Robustness: User authenticated key agreement schemes should withstand different types of attacks, such as off-line password guessing attacks, replay attacks, insider attacks and impersonation attacks.

Furthermore, the efficiency aspect should be considered when applying the authentication mechanism to the WSN environment because the sensor nodes are limited in terms of their computing resources and power [5]. In other words, when constructing an authentication mechanism for WSNs, a hash function-based method is recommended for use since it requires less computation overhead than public-key cryptosystems, such as RSA, elliptic curves cryptography (ECC) and El-gamal, all of which have a high computational overhead [6,7]. Therefore, the authentication protocol implemented for WSNs should be simple and efficient while also conforming to the required security.

### 1.1. Related Studies

In 2006, Wong et al. [8] first presented a lightweight user authentication protocol for WSNs. Their protocol improved the efficiency by only employing a one-way hash function and exclusive-OR operation. However, Das [9] pointed out that Wong et al.’s scheme [8] could not withstand many logged-in users with the same login identity attacks and stolen-verifier attacks. Das [9] then suggested an improved version that solved the flaws present in Wong et al.’s method. Unfortunately, Khan and Alghathbar [10] demonstrated in 2010 that Das’s scheme [9] could not withstand a privileged-insider attack and gateway node bypass attack and proposed an enhanced new strategy. In the same year, Chen and Shih [11] also demonstrated that Das’s scheme [9] overlooks parallel session attacks and cannot support a mutual authentication property. Chen and Shih [11] then proposed an enhanced version. In 2012, Vaidya et al. [12] pointed out that Das’s scheme [9], Khan and Alghathbar’s scheme [10] and Chen and Shih’s scheme [11] contained the same vulnerabilities against a lost smart card attack and sensor node impersonation attack. To compensate for these defects, Vaidya et al. [12] suggested their own authentication scheme, arguing that it can withstand various attack types. However, Kim et al. [13] proved in 2014 that Vaidya et al.’s scheme [12] has some weaknesses, such as to user impersonation attacks and gateway node bypass attacks, and thus proposed an upgraded scheme. In 2015, Chang et al. [14] demonstrated that Kim et al.’s scheme [13] could not prevent an impersonation attack, lost smart card attack or man-in-the-middle attack, and it did not provide session key security. Chang et al. [14] then proposed an improved scheme. However, Park and Park [15] pointed out recently that Chang et al’s scheme [14] still had some weaknesses, such as off-line password guessing attack, perfect forward secrecy problem and incorrectness of password change, and proposed an enhanced new version.

In particular, various cryptography techniques were employed in their protocols in order to improve the security for WSNs. Lee [16] and Kumari et al. [17] apply a chaotic map technique in their authentication mechanism. In 2015, Cheng et al. [18] presented an RSA-based authentication method for WSNs. In addition, Yeh et al. [19] proposed an authentication protocol based on elliptic curves cryptography (ECC) for WSNs. However, Han [20] pointed out that Yeh et al.’s scheme [19] could not achieve perfect forward secrecy and fails to provide mutual authentication. To address these weaknesses, Shi and Gong [21] presented a new authentication mechanism for WSNs using an ECC technique. However, Choi et al. [22] demonstrated that Shi and Gong’s mechanism [21] could not satisfy security requirements because their scheme is unsafe against lost smart card attacks and does not provide session key security.

### 1.2. Motivations and Contributions

In 2015, Chang et al. [14] presented a two-factor user authenticated key agreement scheme for WSNs. They claimed that their scheme could resist an off-line password guessing attack and an impersonation attack, as well as provide session key security. However, we have discovered that Chang et al.’s scheme [14] comprises critical security weaknesses. Their scheme (i) still cannot guarantee protection against an off-line password guessing attack or user impersonation attack, (ii) fails to provide session key security, (iii) is faced with a scalability problem because the gateway nodes in their scheme always maintain verifier tables (iv) and cannot provide session key verification processes.

Our main contribution in this study is as follows. First, we concretely explain the weaknesses in Chang et al.’s scheme. Second, we propose a more developed authentication protocol for WSNs. Third, we show that the proposed mechanism satisfies various security requirements. Finally, we demonstrate that the proposed protocol has better performance than other related studies in terms of the computation cost and time consumption.

### 1.3. Preliminaries

In this subsection, we first introduce the biohash function [23], which is used in our proposed scheme. Then, we list the notations of Chang et al.’s scheme [14] and our proposed scheme in Table 1.

#### 1.3.1. Biohash Function

**Figure 2:**
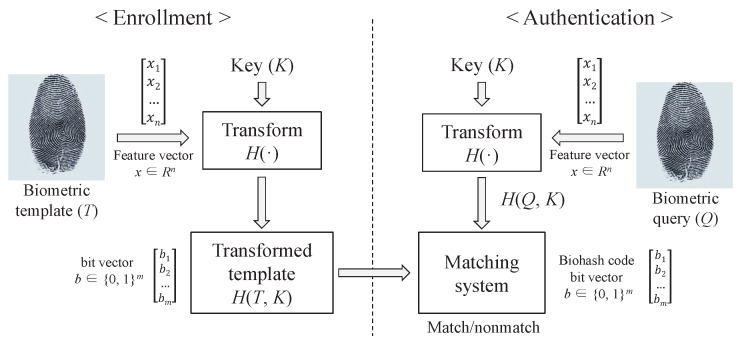
Authentication mechanism using the biohashing approach.

The user’s biometric information is very sensitive data. Thus, when user identification is carried out using biometric data, a secure and sophisticated matching technique is required. In order to handle this concern, in 2004, Jin et al. [23] presented a fingerprint-based function to identify the user’s legitimacy. The biohash technique employs the particular tokenized pseudo-random numbers to each of the users measuring biometric feature arbitrarily onto two-fold strands. Figure 2 describes the user recognition mechanism employing the user’s biometric information and biohashing technique. When a device recognizes user’s biometric template *T*, it transforms *T* into the form of feature vector and then transmits to transform function H(·). Transform function H(·) creates transformed template H(T,K) by inputting the transmitted template *T* and random key *K*. Furthermore, the device creates biohash code, H(Q,K) from the random key *K* and the stored value, which is a biometric query, in order to judge whether the user is registered or not, comparing to the new value, H(T,K). The biohashing technique is also applied in our scheme, illustrated in Section 5. We use an input value Bio as a combination of the user’s biometric information and a random key for convenience, like other authentication schemes [24,25,26,27] using the biohashing technique.

The biohash function H(·) is a one-way function with a feature that can reduce the probability of the denial of service. That is to say, the identical biometric information creates the identical value of H(Bio), and it is impossible to calculate an input value Bio from the result value of H(Bio). Until now, many authentication studies have been conducted [24,25,26,27] based on the biohashing technique. Our proposed scheme also adopts the user’s biometric information applying a biohashing, and the details are given below in Section 5.

#### 1.3.2. Scalability and Practicability in Terms of Authentication Using Biometric Information

The three-factor authentication protocol has been frequently employed in recent days, which complements the two-factor authentication protocol using the identity and password by adding biometric information. Basically, an authentication mechanism using biometric information requires a smart card terminal capable of recognizing a smart card and a device capable of recognizing the user’s biometric (fingerprint) information. To reduce this inconvenience, Baratelli [28] and Kozlay [29] devised a new smart card-based fingerprint identification technology by adding a fingerprint recognition device in the smart card, and Clancy [30] proposed a self-fingerprint authentication technique using a smart card. In other words, a new device that combines a smart card terminal and a fingerprint reader has already been developed. In fact, authentication research does not really mean the inconvenience of fingerprint terminal devices and assumes that devices that can recognize both smart cards and fingerprints are used. In addition, a number of research works with respect to three-factor authentication protocol already [24,25,26,27] have applied user’s biometric information.

First of all, the most important reason for using biometric information in the authentication mechanism is to increase the security of the protocol by preventing identity/password guessing attack. For this reason, our proposed scheme also uses the biometric information of the user, and it is confirmed that the proposed scheme is very safe. A detailed description of the protocol can be found in Section 4, and a security analysis can be found in Section 5.

#### 1.3.3. Notations

The notations used in this paper are listed in Table 1.

### 1.4. Organization of the Paper

The remainder of this paper is structured as follows. In Section 2, we briefly explain Chang et al.’s authentication scheme. Section 3 demonstrates the vulnerabilities in Chang et al.’s scheme. A detailed explanation of our proposed scheme is provided in Section 4. In Section 5, we evaluate whether our proposed scheme can withstand various attacks. Further, we conduct a formal security proof using the random oracle model in Section 6. In Section 7, we analyze the performance of the proposed scheme, and in Section 8, we provide the conclusion to the paper.

## 2. Review of Chang et al.’s Scheme

In this section, we briefly review Chang et al.’s authenticated key agreement scheme [14] to then cryptanalyze their scheme. It is composed of four phases: registration, login, authentication and password change. In Chang et al.’s scheme [14], there are three communication parties, including a user $U_{i}$, a gateway node GWN and a sensor node $S_{j}$. We describe each phase in detail, and Table 1 shows the notations used in Chang et al.’s scheme.

### 2.1. Registration Phase

(1) $U_{i}$ selects $ID_{i}$ and $PW_{i}$, and $U_{i}$ then generates a random number $RN_{r}$. $U_{i}$ computes $HPW_{i}=h(PW_{i}||RN_{r})$ and sends a registration request $\langle ID_{i},HPW_{i}\rangle$ to GWN through a secure channel.
(2) GWN computes $HID_{i}=h(ID_{i}\left||K\right)$, $X_{S_{i}}=h(HID_{i}\left||K\right)$, $A_{i}=h(HPW_{i}\left|\right|X_{S_{i}})⊕HID_{i}$, $B_{i}=h\left(HPW_{i}⊕X_{S_{i}}\right)$ and $C_{i}=X_{S_{i}}⊕h(ID_{s}||HPW_{i})$ and maintains $\left(TID_{i},TID_{i}^{\circ },HID_{i}\right)$ in its database for $U_{i}$, where $TID_{i}=RN_{G}$ and $TID_{i}^{\circ }{=}^{\prime \prime \prime \prime }$. GWN chooses a smart card and writes $\left\{ID_{s},A_{i},B_{i},C_{i},TID_{i},h\left(\cdot \right)\right\}$ into the smart card’s memory. Then, GWN sends the smart card to $U_{i}$ through a secure channel.
(3) $U_{i}$ computes $XPW_{i}=h\left(PW_{i}\right)⊕RN_{r}$ and stores $XPW_{i}$ in the smart card’s memory. Finally, the smart card contains the information $\left\{ID_{s},A_{i},B_{i},C_{i},TID_{i},h\left(\cdot \right),XPW_{i}\right\}$.

### 2.2. Login Phase

(1) $U_{i}$ inserts $U_{i}$’s smart card into a terminal and inputs the $ID_{i}$ and $PW_{i}$. The smart card computes $RN_{r}^{*}=h\left(PW_{i}\right)⊕XPW_{i}$, $HPW_{i}^{*}=h(PW_{i}||RN_{r}^{*})$, $X_{S_{i}}^{*}=C_{i}⊕h(ID_{s}||HPW_{i}^{*})$, $B_{i}^{*}=h\left(HPW_{i}^{*}⊕X_{S_{i}}^{*}\right)$ and compares $B_{i}^{*}$ with the stored value $B_{i}$. If this condition is satisfied, the smart card acknowledges the legitimacy of $U_{i}$ and proceeds with the next step. Otherwise, it terminates this phase.
(2) The smart card computes $k_{i}=h(X_{S_{i}}^{*}\left|\right|T_{1})$, $DID_{i}=h(HPW_{i}^{*}\left|\right|X_{S_{i}}^{*})⊕k_{i}$ and $M_{U_{i},G}=h(A_{i}\left|\right|X_{S_{i}}^{*}\left|\right|T_{1})$.
(3) Finally, $U_{i}$ sends a login request $\langle DID_{i},M_{U_{i},G},T_{1},TID_{i}\rangle$ to GWN through a public channel.

### 2.3. Authentication Phase

(1) GWN first checks the validity of the time stamp $|T_{1}^{\prime }-T_{1}|<\Delta T$ and retrieves $HID_{i}$ from $TID_{i}$ corresponding to $TID_{i}$ in its database. If GWN cannot search the $TID_{i}$, GWN retrieves $HID_{i}$ from $TID_{i}^{\circ }$. GWN, then computes $X_{S_{i}}=h(HID_{i}\left||K\right)$, $k_{i}=h(X_{S_{i}}\left|\right|T_{1})$, $X^{*}=DID_{i}⊕k_{i}$, $M_{U_{i},G}^{*}=h(X^{*}⊕HID_{i}\left|\right|X_{S_{i}}\left|\right|T_{i})$ and compares $M_{U_{i},G}^{*}$ with the received value $M_{U_{i},G}$. If this condition is satisfied, GWN acknowledges the legitimacy of the $U_{i}$ and proceeds with the next step. Otherwise, it terminates this phase.
(2) GWN computes $X_{S_{j}}=h(SID_{j}\left||K\right)$, $M_{G,S_{j}}=h(DID_{i}||SID_{j}\left|\right|X_{S_{j}}\left|\right|T_{2})$, then sends the message $\langle DID_{i},M_{G,S_{j}},T_{2}\rangle$ to $S_{j}$ through a public channel.
(3) $S_{j}$ checks whether $|T_{2}^{\prime }-T_{2}|<\Delta T$. $S_{j}$ then computes $M_{G,S_{j}}^{*}=h(DID_{i}||SID_{j}\left|\right|X_{S_{j}}^{*}\left|\right|T_{2})$ and compares $M_{G,S_{j}}^{*}$ with the received value $M_{G,S_{j}}$. If this condition is satisfied, $S_{j}$ believes that the GWN is authentic. Otherwise, it terminates this phase.
(4) $S_{j}$ computes $k_{j}=h(X_{S_{j}}^{*}\left|\right|T_{3})$, $z_{i}=M_{G,S_{j}}^{*}⊕k_{j}$, $K_{S}=f\left(DID_{i},k_{j}\right)$, $M_{S_{j},G}=h(z_{i}\left|\right|X_{S_{j}}^{*}\left|\right|T_{3})$ and then sends the message $\langle M_{S_{j},G},T_{3}\rangle$ to GWN through a public channel.
(5) GWN checks whether $|T_{3}^{\prime }-T_{3}|<\Delta T$. GWN then computes $k_{j}=h(X_{S_{j}}\left|\right|T_{3})$, $z_{i}^{*}=M_{G,S_{j}}⊕k_{j}$, $M_{S_{j},G}^{*}=h(z_{i}^{*}\left|\right|X_{S_{j}}\left|\right|T_{3})$ and compares $M_{S_{j},G}^{*}$ with the received value $M_{S_{j},G}$. If true, GWN believes that the $S_{j}$ is authentic. Otherwise, GWN terminates this phase.
(6) GWN computes $M_{G,U_{i}}=h(DID_{i}\left|\right|M_{U_{i},G}^{*}\left|\right|k_{j}\left|\right|X_{S_{i}}\left|\right|T_{4})$, $y_{i}=k_{j}⊕h\left(k_{i}\right)$, $TID_{i\_new}=h(HID_{i}\left|\right|T_{1})$ and updates $\left(TID_{i},TID_{i}^{\circ }\right)$ as $\left(TID_{i\_new},TID_{i}\right)$ in its database. GWN then sends the message $\langle y_{i},M_{G,U_{i}},T_{4}\rangle$ to $U_{i}$ through a public channel.
(7) $U_{i}$ checks whether $|T_{4}^{\prime }-T_{4}|\leq \Delta T$. $U_{i}$ then computes $k_{j}^{*}=y_{i}⊕h\left(k_{i}\right)$, $M_{G,U_{i}}^{*}=h(DID_{i}\left|\right|M_{U_{i},G}\left|\right|k_{j}^{*}\left|\right|X_{S_{i}}\left|\right|T_{4})$ and compares $M_{G,U_{i}}^{*}$ with the received value $M_{G,U_{i}}$. If the verification does not hold, this phase is terminated. Otherwise, $U_{i}$ believes that the GWN is authentic and computes the shared session key $K_{S}=f\left(DID_{i},k_{j}^{*}\right)$.
(8) $U_{i}$ computes $HID_{i}=A_{i}⊕h(HPW_{i}^{*}\left|\right|X_{S_{i}}^{*})$ and $h(HID_{i}||T_{1})$. Lastly, $U_{i}$ updates $TID_{i}$ as $h(HID_{i}||T_{1})$ and successfully ends the authentication phase.

### 2.4. Password Change Phase

(1) $U_{i}$ inserts $U_{i}$’s smart card into a card reader and inputs $ID_{i}$, the old password $PW_{i}$ and new password $PW_{i}^{new}$. The smart card computes $RN_{r}^{*}=h\left(PW_{i}\right)⊕XPW_{i}$, $HPW_{i}^{*}=h(PW_{i}||RN_{r}^{*})$, $X_{S_{i}}^{*}=C_{i}⊕h(ID_{s}||HPW_{i}^{*})$, $B_{i}^{*}=h\left(HPW_{i}^{*}⊕X_{S_{i}}^{*}\right)$ and compares $B_{i}^{*}$ with the stored value $B_{i}$. If this condition is not satisfied, it terminates this phase. Otherwise, the smart card proceeds with the next step.
(2) The smart card computes $HPW_{i}^{new}=h(PW_{i}^{new}||RN_{r}^{*})$, $A_{i}^{new}=h(HPW_{i}^{new}\left|\right|X_{S_{i}})⊕HID_{i}$, $B_{i}^{new}=h\left(HPW_{i}^{new}⊕X_{S_{i}}\right)$$C_{i}^{new}=X_{S_{i}}⊕h(ID_{s}||HPW_{i}^{new})$ and $XPW_{i}^{new}=h\left(PW_{i}^{new}\right)⊕RN_{r}$.
(3) The smart card replaces the existing value $\left(A_{i},B_{i},C_{i},XPW_{i}\right)$ with the new values $\left(A_{i}^{new},B_{i}^{new},C_{i}^{new},XPW_{i}^{new}\right)$.

## 3. Security Weaknesses of Chang et al.’s Scheme

In this section, we show that Chang et al.’s scheme [14] possesses a number of security vulnerabilities. The following vulnerabilities are based on the two assumptions that

- An attacker can extract all parameters stored in the smart card by physically monitoring its power consumption [31].
- An attacker can eavesdrop or reform any messages in the public channel [32,33].

Under these two assumptions, the following problems have been found, and their detailed descriptions are given below.

### 3.1. Off-Line Password Guessing Attack

This attack attempts to input a password until the correct password is discovered because many users have a tendency to employ simple, brief passwords for the sake of convenience. For this reason, the authentication mechanism for all passwords should be invented to guarantee protection against a guessing attack. However, Chang et al.’s scheme [14] has a weakness in this situation, and we therefore propose a scenario for an off-line password-guessing attack. The following is a detailed description:

Step 1. An attacker extracts $\left\{ID_{s},A_{i},B_{i},C_{i},TID_{i},h\left(\cdot \right),XPW_{i}\right\}$ from $U_{i}$’s stolen smart card by physically monitoring its power consumption [31].
Step 2. The attacker collects a valid login request $\langle DID_{i},M_{U_{i},G},T_{1},TID_{i}\rangle$ from the previous session [32,33].
Step 3. The attacker selects a password candidate $PW_{i}^{*}$.
Step 4. The attacker computes $HPW_{i}^{*}=h(PW_{i}^{*}||h\left(PW_{i}^{*}\right)⊕XPW_{i})$ using the password candidate $PW_{i}^{*}$.
Step 5. The attacker then computes:
  $$\begin{array}{ccc} X_{S_{i}}^{*} & = & C_{i}⊕h(ID_{s}||HPW_{i}^{*})\\ & = & C_{i}⊕h(ID_{s}||h(PW_{i}^{*}||h\left(PW_{i}^{*}\right)⊕XPW_{i}))\\ B_{i}^{*} & = & h(HPW_{i}^{*}⊕X_{S_{i}}^{*})\\ & = & h(h(PW_{i}^{*}||h\left(PW_{i}^{*}\right)⊕XPW_{i})⊕C_{i}⊕h(ID_{s}||h(PW_{i}^{*}||h\left(PW_{i}^{*}\right)⊕XPW_{i}))) \end{array}$$
Step 6. The attacker repeats the steps above from 3–5 until the computed result $B_{i}^{*}$ equals the breached secret $B_{i}$.
Step 7. If they correspond with each other, $PW_{i}^{*}$ would be an accurate password. If not, the attacker repeats the above steps until the correct password is found.

Therefore, we can realize that Chang et al.’s scheme [14] is vulnerable to the off-line password guessing attack.

### 3.2. User Impersonation Attack

The security of the password-based authentication mechanism relies on the complexity of the password. Thus, if an attacker obtains a password, the attacker can pretend to be a legal user. Unfortunately, Chang et al.’s scheme [14] allows an attacker to impersonate a legal user if the attacker obtains the user’s password $PW_{i}$ through a guessing attack. The following is a detailed description of this scenario:

Step 1. An attacker extracts $\left\{ID_{s},A_{i},B_{i},C_{i},TID_{i},h\left(\cdot \right),XPW_{i}\right\}$ from $U_{i}$’s stolen smart card [31].
Step 2. The attacker collects a valid login request $\langle DID_{i},M_{U_{i},G},T_{1},TID_{i}\rangle$ from the previous session.
Step 3. The attacker obtains the user’s $PW_{i}$ through an off-line password guessing attack.
Step 4. The smart card computes:
  $$\begin{array}{ccc} DID_{i}^{*} & = & h(HPW_{i}\left|\right|X_{S_{i}})⊕k_{i}\\ & = & h(HPW_{i}\left|\right|X_{S_{i}})⊕h(X_{S_{i}}\left|\right|T_{1}),\mathrm{where}\;X_{S_{i}}=C_{i}⊕h(ID_{s}||h(PW_{i}||h\left(PW_{i}\right)⊕XPW_{i}))\\ M_{U_{i},G}^{*} & = & h(A_{i}||X_{S_{i}}||T_{1})\\ & = & h(A_{i}\left|\right|C_{i}⊕h(ID_{s}||h(PW_{i}||h\left(PW_{i}\right)⊕XPW_{i}))||T_{1}) \end{array}$$
Step 5. The attacker then sends a counterfeited login request $\langle DID_{i}^{*},M_{U_{i},G}^{*},T_{1},TID_{i}\rangle$ to GWN through a public channel.
Step 6. After receiving the $\langle DID_{i}^{*},M_{U_{i},G}^{*},T_{1},TID_{i}\rangle$, GWN computes $X_{S_{i}}=h(HID_{i}\left||K\right)$, $k_{i}=h(X_{S_{i}}\left|\right|T_{i})$, $X=DID_{i}^{*}⊕k_{i}$ and $M_{U_{i},G}=h(X⊕HID_{i}\left|\right|X_{S_{i}}\left|\right|T_{i})$.
Step 7. GWN compares the computed value $M_{U_{i},G}$ with the received value $M_{U_{i},G}^{*}$. Finally, GWN successfully finishes the verification process because $M_{U_{i},G}^{*}$, which is computed by the attacker, is correctly equal to $M_{U_{i},G}$, which is computed by the GWN.

Through the aforementioned descriptions, the attacker can successfully pass the checking process and be disguised as a legal user under Chang et al.’s scheme [14].

### 3.3. Session Key Compromise

In Chang et al.’s scheme [14], if an attacker knows $U_{i}$’s password $PW_{i}$, the attacker can establish the session key $K_{S}=f\left(DID_{i},k_{j}\right)$ shared between $U_{i}$ and $S_{j}$. First, the attacker can extract $\left\{ID_{s},A_{i},B_{i},C_{i},TID_{i},h\left(\cdot \right),XPW_{i}\right\}$ from $U_{i}$’s stolen smart card. Second, the attacker can obtain $DID_{i}$ and $y_{i}$ after eavesdropping on the messages $\langle DID_{i},M_{U_{i},G},T_{1},TID_{i}\rangle$ and $\langle y_{i},M_{G,U_{i}},T_{4}\rangle$. Then, the attacker can try to compute $k_{j}=y_{i}⊕h\left(k_{i}\right)=y_{i}⊕h(h(C_{i}⊕h(ID_{s}||h(PW_{i}||h\left(PW_{i}\right)⊕XPW_{i}))||T_{1}))$ using the acquired $PW_{i}$, which has been previously compromised as in Section 3.1. With the combined $\left\{y_{i},C_{i},ID_{s},PW_{i},XPW_{i},T_{1}\right\}$ values, the attacker can successfully construct the $K_{S}=f\left(DID_{i},k_{j}\right)$.

### 3.4. Scalability Problem

In order to provide convenience, Chang et al. [14] suggested that the GWN maintains a verifier table in the database to save the information, such as the user’s temporary identities $\left(TID_{i},TID_{i}^{\circ }\right)$ and $HID_{i}=h(ID_{i}\left||K\right)$ value. Accordingly, the GWN should always need to retain each user’s verifier table. However, the increased amount of user information that needs to be retained places greater burden on the GWN since the number of verifier tables will increase as the number of users’ increases. Moreover, the use of the verifier table is inefficient in terms of the computation time since the changed values at each phase need to be updated in the verifier table.

### 3.5. Absence of a Session Key Verification Process

According to [34,35], the authenticated key agreement mechanism recommends a verification procedure to verify the coherence of the generated session keys between the communicating parties. In the authentication phase in Chang et al.’s scheme [14], $U_{i}$ generates his/her own session key $K_{S}$ after verifying the message $\langle y_{i},M_{G,U_{i}},T_{4}\rangle$ through $M_{G,U_{i}}^{*}\overset{?}{=}M_{G,U_{i}}$. However, in this case, because of the $M_{S_{j},G}=h(z_{i}\left|\right|X_{S_{j}}^{*}\left|\right|T_{3})$ has no information about the session key generated by $S_{j}$, and the $U_{i}$ can hardly be sure whether a new generated session key $K_{S}$ is precisely the same as the $S_{j}$’s session key or not. Therefore, the following procedures [34] are required to ensure an accurate session key distribution between a $U_{i}$ and a $S_{j}$: (1) after generating a session key, $S_{j}$ sends a message, including information regarding the generated session key; (2) the $U_{i}$ should guarantee the accuracy of the session key from the $S_{j}$, verifying the received message.

## 4. The Proposed Scheme

**Figure 3:**
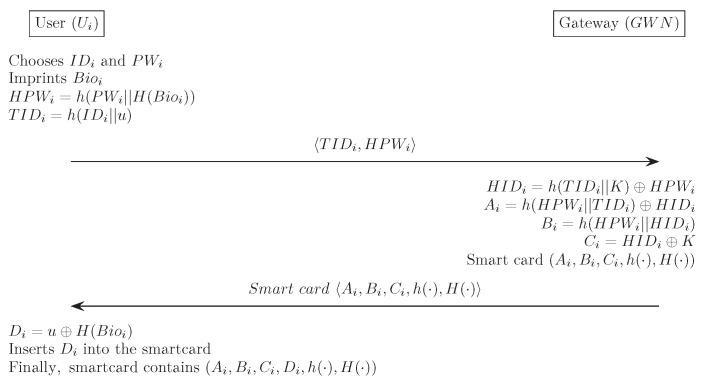
Registration phase for the proposed scheme.

**Figure 4:**
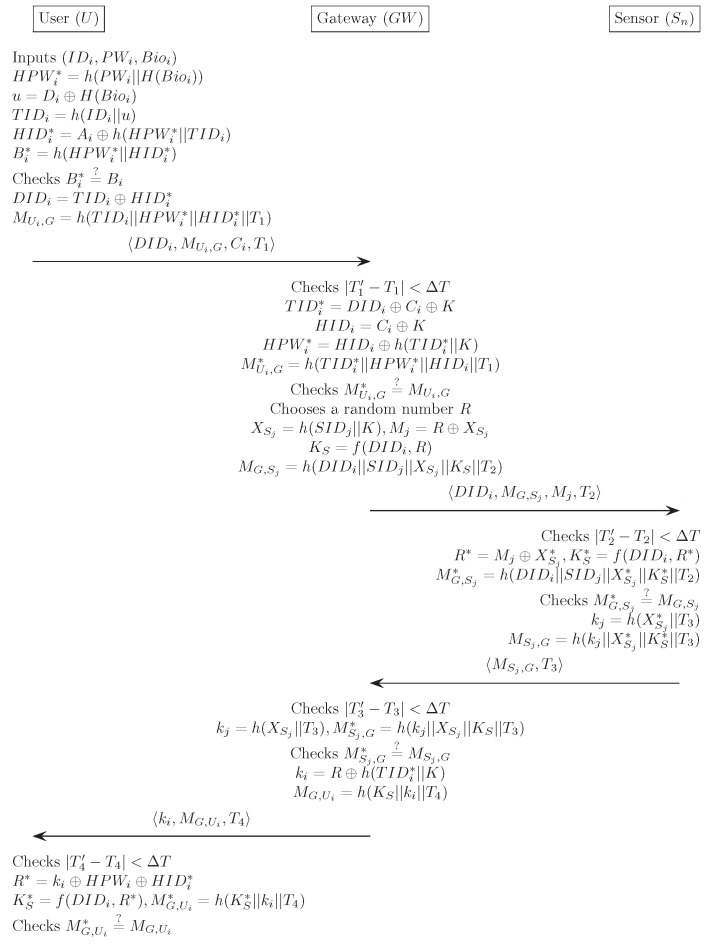
Login and authentication phase for the proposed scheme.

**Figure 5:**
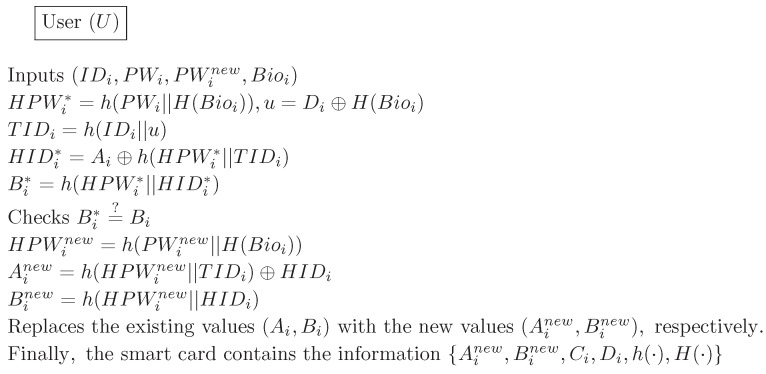
Password change phase for the proposed scheme.

In this section, we suggest an improved version of the authenticated key agreement mechanism for the WSN in order to provide improved security by resolving Chang et al.’s [14] weaknesses. In the proposed scheme, to guarantee protection from the off-line password guessing attack, we employ biometrics information with the biohashing technique H(·) [23], as mentioned in Section 1.3. By preventing an off-line password guessing attack, our scheme can guarantee protection against an impersonation attack and against session key compromise. In addition, we remove the verifier table stored in GWN to increase efficiency. Our proposed scheme also consists of four phases: registration, login, authentication and password change. We describe each phase in detail, and Figure 3, Figure 4 and Figure 5 describe our scheme. The notation used in the proposed scheme is displayed in Table 1.

### 4.1. Registration Phase

The registration phase begins when the $U_{i}$ sends a request message for registration to GWN through a secure channel. The GWN then issues a smart card, including some information, and sends it to $U_{i}$. Meanwhile, $S_{j}$ stores pre-defined values $SID_{j}$ and $X_{S_{j}}^{*}$ in its memory, where $X_{S_{j}}^{*}=h(SID_{j}\left||K\right)$. The following describes this process in detail, and Figure 2 illustrates the registration phase for our proposed scheme.

(1) $U_{i}$ selects $ID_{i}$ and $PW_{i}$, and $U_{i}$ then imprints his/her biometrics $Bio_{i}$. $U_{i}$ computes $HPW_{i}=h(PW_{i}||H\left(Bio_{i}\right))$, generates a random number *u* and computes $TID_{i}=h(ID_{i}\left||u\right)$. $U_{i}$ sends a registration request $\langle TID_{i},HPW_{i}\rangle$ to GWN through a secure channel.
(2) GWN computes $HID_{i}=h(TID_{i}\left||K\right)⊕HPW_{i}$, $A_{i}=h(HPW_{i}||TID_{i})⊕HID_{i}$, $B_{i}=h(HPW_{i}||HID_{i})$ and $C_{i}=HID_{i}⊕K$. GWN chooses a smart card and writes $\left\{A_{i},B_{i},C_{i},h\left(\cdot \right),H\left(\cdot \right)\right\}$ into the smart card’s memory. Then, GWN sends the smart card to $U_{i}$ through a secure channel.
(3) Upon receiving the smart card, $U_{i}$ computes $D_{i}=u⊕H\left(Bio_{i}\right)$ and stores it in the smart card. Finally, the smart card contains the information $\left\{A_{i},B_{i},C_{i},D_{i},h\left(\cdot \right),H\left(\cdot \right)\right\}$.

### 4.2. Login Phase

The login phase is executed whenever the $U_{i}$ wants to gain access to WSN using his/her $ID_{i}$, $PW_{i}$ and smart card. In this phase, $U_{i}$ sends the login request to GWN. Figure 3 illustrates the login and authentication phase for our proposed scheme. The following describes this process in detail.

(1) $U_{i}$ inserts $U_{i}$’s smart card into a terminal and inputs the $ID_{i}$, $PW_{i}$ and imprints biometric $Bio_{i}$. The smart card computes $HPW_{i}^{*}=h(PW_{i}||H\left(Bio_{i}\right))$, $u=D_{i}⊕H\left(Bio_{i}\right)$, $TID_{i}=h(ID_{i}\left||u\right)$, $HID_{i}^{*}=A_{i}⊕h(HPW_{i}^{*}||TID_{i})$, $B_{i}^{*}=h(HPW_{i}^{*}||HID_{i}^{*})$ and compares $B_{i}^{*}$ with the stored value $B_{i}$. If this condition is satisfied, the smart card acknowledges the legitimacy of the $U_{i}$ and proceeds to the next step. Otherwise, it terminates this phase.
(2) The smart card computes $DID_{i}=TID_{i}⊕HID_{i}^{*}$ and $M_{U_{i},G}=h(TID_{i}||HPW_{i}^{*}||HID_{i}^{*}\left|\right|T_{1})$.
(3) Finally, $U_{i}$ sends a login request $\langle DID_{i},M_{U_{i},G},C_{i},T_{1}\rangle$ to GWN through a public channel.

### 4.3. Authentication Phase

The authentication phase begins when GWN receives the login request from the $U_{i}$. This phase performs several steps to achieve mutual authentication, as well as a session key agreement between $U_{i}$, GWN and $S_{j}$ involved within the WSN. The following describes this process in detail.

(1) GWN first checks the validity of the time stamp $|T_{1}^{\prime }-T_{1}|<\Delta T$ and computes $TID_{i}^{*}=DID_{i}⊕C_{i}⊕K$, $HID_{i}=C_{i}⊕K$ and $HPW_{i}^{*}=HID_{i}⊕h(TID_{i}^{*}\left||K\right)$. GWN further computes $M_{U_{i},G}^{*}=h(TID_{i}^{*}||HPW_{i}^{*}||HID_{i}\left|\right|T_{1})$ and compares it with the received value $M_{U_{i},G}$. If this condition is satisfied, GWN acknowledges the legitimacy of the $U_{i}$ and proceeds with the next step. Otherwise, it terminates this phase.
(2) GWN generates a random number *R* and computes $X_{S_{j}}=h(SID_{j}\left||K\right)$, $M_{j}=R⊕X_{S_{j}}$, $K_{S}=f\left(DID_{i},R\right)$ and $M_{G,S_{j}}=h(DID_{i}||SID_{j}\left|\right|X_{S_{j}}\left|\right|K_{S}\left|\right|T_{2})$. GWN then sends the message $\langle DID_{i},M_{G,S_{j}},M_{j},T_{2}\rangle$ to $S_{j}$ through a public channel.
(3) $S_{j}$ checks whether $|T_{2}^{\prime }-T_{2}|<\Delta T$ and computes $R^{*}=M_{j}⊕X_{S_{j}}^{*}$ and $K_{S}^{*}=f\left(DID_{i},R^{*}\right)$. $S_{j}$ further computes $M_{G,S_{j}}^{*}=h(DID_{i}||SID_{j}\left|\right|X_{S_{j}}^{*}\left|\right|K_{S}^{*}\left|\right|T_{2})$ and compares it with the received value $M_{G,S_{j}}$. If this condition is satisfied, $S_{j}$ believes that the GWN is authentic. Otherwise, it terminates this phase.
(4) $S_{j}$ computes $k_{j}=h(X_{S_{j}}^{*}\left|\right|T_{3})$ and $M_{S_{j},G}=h(k_{j}\left|\right|X_{S_{j}}^{*}\left|\right|K_{S}^{*}\left|\right|T_{3})$. $S_{j}$ then sends the message $\langle M_{S_{j},G},T_{3}\rangle$ to GWN through a public channel.
(5) GWN checks whether $|T_{3}^{\prime }-T_{3}|<\Delta T$. GWN computes $k_{j}=h(X_{S_{j}}\left|\right|T_{3})$, $M_{S_{j},G}^{*}=h(k_{j}\left|\right|X_{S_{j}}\left|\right|K_{S}\left|\right|T_{3})$ and compares $M_{S_{j},G}^{*}$ with the received value $M_{S_{j},G}$. If true, GWN believes that the $S_{j}$ is authentic. Otherwise, GWN terminates this phase.
(6) GWN computes $k_{i}=R⊕h(TID_{i}^{*}\left||K\right)$ and $M_{G,U_{i}}=h(K_{S}\left|\right|k_{i}\left|\right|T_{4})$. GWN then sends the message $\langle k_{i},M_{G,U_{i}},T_{4}\rangle$ to $U_{i}$ through a public channel.
(7) $U_{i}$ checks whether $|T_{4}^{\prime }-T_{4}|\leq \Delta T$ and computes $R^{*}=k_{i}⊕HPW_{i}⊕HID_{i}^{*}$ and $K_{S}^{*}=f\left(DID_{i},R^{*}\right)$. $U_{i}$ further computes $M_{G,U_{i}}^{*}=h(K_{S}^{*}\left|\right|k_{i}\left|\right|T_{4})$ and compares it with the received value $M_{G,U_{i}}$. If this condition is not satisfied, this phase is terminated. Otherwise, $U_{i}$ believes that the GWN is authentic and successfully ends the authentication phase

### 4.4. Password Change Phase

The password change phase begins when the $U_{i}$ intends to change the original password $PW_{i}$ to a new password $PW_{i}^{new}$. Figure 4 illustrates the password change phase for our proposed scheme. The following describes this process in detail.

(1) $U_{i}$ inserts $U_{i}$’s smart card into a terminal, inputs $ID_{i}$, $PW_{i}$, $PW_{i}^{new}$ and then imprints biometric $Bio_{i}$. The smart card computes $HPW_{i}^{*}=h(PW_{i}||H\left(Bio_{i}\right))$, $u=D_{i}⊕H\left(Bio_{i}\right)$, $TID_{i}=h(ID_{i}\left||u\right)$, $HID_{i}^{*}=A_{i}⊕h(HPW_{i}^{*}||TID_{i})$, $B_{i}^{*}=h(HPW_{i}^{*}||HID_{i}^{*})$ and compares $B_{i}^{*}$ with the stored value $B_{i}$. If this condition is not satisfied, it terminates this phase. Otherwise, the smart card proceeds with the next step.
(2) The smart card computes $HPW_{i}^{new}=h(PW_{i}^{new}||H\left(Bio_{i}\right))$, $A_{i}^{new}=h(HPW_{i}^{new}||TID_{i})⊕HID_{i}$ and $B_{i}^{new}=h(HPW_{i}^{new}||HID_{i})$.
(3) The smart card replaces the existing values $A_{i}$ and $B_{i}$ with the new values $A_{i}^{new}$ and $B_{i}^{new}$, respectively. Finally, the smart card contains the information $\left\{A_{i}^{new},B_{i}^{new},C_{i},D_{i},h\left(\cdot \right),H\left(\cdot \right)\right\}$.

## 5. Security Analysis and Proof of the Proposed Scheme

In this section, we first describe whether the proposed scheme can withstand various attacks and also satisfy the basic requirements. Moreover, we adopt Burrows–Abadi–Needham (BAN) logic [36] to prove that a session key can be correctly generated between $U_{i}$ and $S_{j}$. The results are described as follows.

### 5.1. Informal Security Analysis of the Proposed Scheme

In this subsection, our proposed scheme is examined against various attacks and is evaluated according to the suitability of the basic requirements [5,6,7,8,9,10,11,12,13]. We also conduct a comparative analysis [10,12,13,14,15], which is illustrated in Table 2.

• The proposed scheme preserves user anonymity:

User anonymity is a valuable property for the user authentication protocol because the exposure of a user’s identity can allow an unauthorized party to track the user’s login pattern. Suppose that the attacker has intercepted $U_{i}$’s login request $\langle DID_{i},M_{U_{i},G},C_{i},T_{1}\rangle$ and extracted information $\left\{A_{i},B_{i},C_{i},D_{i},h\left(\cdot \right),H\left(\cdot \right)\right\}$ in a stolen smart card [31]. The attacker may then try to compute $ID_{i}$ through $h(ID_{i}\left||u\right)=DID_{i}⊕HID_{i}$. However, it is impossible to know $HID_{i}$ since $HID_{i}$ consists of $\left(C_{i}⊕K\right)$ and the secret key *K* is only known to GWN. In addition, *u* includes $H(Bio_{i})$ information that is only known to $U_{i}$. Therefore, the attacker cannot acquire the user’s $ID_{i}$.

• The proposed scheme achieves mutual authentication:

In the authentication phase of our scheme, $U_{i}$, GWN and $S_{j}$ authenticate each other through some checking processes. In detail, GWN first verifies the login request $\langle DID_{i},M_{U_{i},G},C_{i},T_{1}\rangle$ by checking whether $M_{U_{i},G}^{*}$ = $M_{U_{i},G}$. $S_{j}$ also verifies the message $\langle DID_{i},M_{G,S_{j}},M_{j},T_{2}\rangle$ by checking whether $M_{G,S_{j}}^{*}$ = $M_{G,S_{j}}$. In addition, GWN and $U_{i}$ verify the messages $\langle M_{S_{j},G},T_{3}\rangle$ and $\langle k_{i},M_{G,U_{i}},T_{4}\rangle$ by checking $M_{S_{j},G}^{*}\overset{?}{=}M_{S_{j},G}$ and $M_{G,U_{i}}^{*}\overset{?}{=}M_{G,U_{i}}$, respectively. Thus, all transmitted messages in our scheme are successfully verified, and our scheme can achieve mutual authentication.

• The proposed scheme withstands stolen smart card attacks:

In our scheme, even if an attacker extracts secret values $\left\{A_{i},B_{i},C_{i},D_{i},h\left(\cdot \right),H\left(\cdot \right)\right\}$ stored in a stolen smart card through the power consumption technique [31], the attack cannot lead to other malicious attacks. In order to obtain the $ID_{i}$, the attack has to know the secret key *K* and $H(Bio_{i})$. However, it is impossible to know the *K* and $H(Bio_{i})$. Therefore, if the attacker does not know the user’s $ID_{i}$, the attacker cannot impersonate a legitimate user. Thus, our proposed scheme can withstand a stolen smart card attack.

• The proposed scheme withstands replay attacks:

In our scheme, all transmitted messages include current time stamp values, such as $T_{1},T_{2},T_{3}$ or $T_{4}$. Therefore, even if an attacker intercepts the login request message and tries to login GWN, the attacker cannot pass the time stamp checking process during the authentication phase. Thus, our proposed scheme can withstand a replay attack.

• The proposed scheme withstands off-line password guessing attacks:

An off-line password guessing attack occurs when an attacker attempts to guess a password and eventually finds the exact user’s password in an off-line environment. This comes from the tendency that many users create simple and brief passwords for their personal convenience, which makes the attacker easily acquire the users’ password by guessing the off-line password without a time limit [37]. For these reasons, the authentication schemes for all password-based users should be designed to prevent a guessing attack.

In our scheme, the attacker can obtain $\left\{A_{i},B_{i},C_{i},D_{i},h\left(\cdot \right),H\left(\cdot \right)\right\}$ from the stolen smart card [31] and can intercept the login request $\langle DID_{i},M_{U_{i},G},C_{i},T_{1}\rangle$. Using these values, the attacker may try to guess the correct identity $ID_{i}^{\prime }$ and password $PW_{i}^{\prime }$ through $B_{i}^{\prime }=h(h(PW_{i}^{\prime }||H\left(Bio_{i}\right))||A_{i}⊕h(h(PW_{i}^{\prime }||H\left(Bio_{i}\right)||TID_{i}^{\prime }))$ or $DID_{i}⊕A_{i}=TID_{i}^{\prime }⊕h(h(PW_{i}^{\prime }||H\left(Bio_{i}\right)||TID_{i}^{\prime })$. However, without knowing $Bio_{i}$, the attacker cannot guess $PW_{i}^{\prime }$. In addition, $H(Bio_{i})$ is hashed biometric information, which is only known by $U_{i}$. Therefore, our proposed scheme is secure against off-line password guessing attacks.

• The proposed scheme withstands user impersonation attacks:

In order to impersonate a legitimate $U_{i}$, the attacker should modify the login request $\langle DID_{i},M_{U_{i},G},C_{i},T_{1}\rangle$ after obtaining the value of $ID_{i}$. However, as we mentioned above, it is impossible for an attacker to obtain the value of $ID_{i}$. Thus, the attacker fails to compute $DID_{i}=TID_{i}⊕HID_{i}$ and cannot generate a sufficient login request to cheat GWN. Therefore, our proposed scheme can withstand a user impersonation attack.

• The proposed scheme withstands sensor node impersonation attacks with node capture:

Suppose that the attacker captures the sensor node $S_{j}$ and extracts information $\left(SID_{j},X_{S_{j}}^{*}\right)$ [13]. The attacker then tries to modify the message $\langle M_{S_{j},G},T_{3}\rangle$ to impersonate a legitimate $S_{j}$. However, the attacker cannot generate a valid message because $X_{S_{j}}^{*}$ consists of $h(SID_{j}||K)$, and it is not feasible to obtain the *K*. Therefore, the attacker cannot impersonate a valid sensor node.

• The proposed scheme provides password verification process:

There is a possibility that a user inputs an incorrect password by mistake. However, for the password verification procedure, the incorrect password will be detected after performing the authentication phase. Our scheme considers this kind of inefficiency situation, verifying the correctness of password $PW_{i}$ by checking the value $B_{i}$ at the beginning of the login phase.

• The proposed scheme provides the session key verification process:

In our scheme, after generating a session key $K_{S}^{*}=f\left(DID_{i},R^{*}\right)$, $S_{j}$ computes $M_{S_{j},G}=h(k_{j}\left|\right|X_{S_{j}}^{*}\left|\right|K_{S}^{*}\left|\right|T_{3})$ and sends the message $\langle M_{S_{j},G},T_{3}\rangle$ to GWN. GWN then computes $k_{i}=R⊕h(TID_{i}^{*}\left||K\right)$ and $M_{G,U_{i}}=h(K_{S}\left|\right|k_{i}\left|\right|T_{4})$, and sends the message $\langle k_{i},M_{G,U_{i}},T_{4}\rangle$ to $U_{i}$. After receiving the message, $U_{i}$ computes $R^{*}=k_{i}⊕HPW_{i}⊕HID_{i}^{*}$, $K_{S}^{*}=f\left(DID_{i},R^{*}\right)$ and $M_{G,U_{i}}^{*}=h(K_{S}^{*}\left|\right|k_{i}\left|\right|T_{4})$ and then compares $M_{G,U_{i}}^{*}$ with the received value $M_{G,U_{i}}$. Since $M_{G,U_{i}}$ includes the information of the session key $K_{S}$, $U_{i}$ may be sure that the $K_{S}$ generated by $S_{j}$ and GWN is accurate if the comparison result $M_{G,U_{i}}^{*}=M_{G,U_{i}}$ is correct. Therefore, our scheme provides a session key verification process.

• The proposed scheme withstands privileged-insider attacks:

An insider attack means that an insider can directly obtain the user’s password from the server and can then access the user’s account in another server by using the same password. During the registration phase of our scheme, $PW_{i}$ is transmitted not as a revealed condition, but as a form of $HPW_{i}=h(PW_{i}||H\left(Bio_{i}\right))$ when $U_{i}$ sends a registration request $\langle TID_{i},HPW_{i}\rangle$ to GWN. Accordingly, the insider attacker in GWN cannot identify the $U_{i}$’s $PW_{i}$. Thus, our scheme can withstand an insider attack.

• The proposed scheme provides session key security:

In our scheme, in order to compromise the session key $K_{S}=f\left(DID_{i},R\right)$, the attacker should know the random number *R*. Therefore, the attacker may try to obtain *R* through $R=M_{j}⊕h(SID_{j}\left||K\right)$. However, it is impossible for an attacker to compute *R* because the attacker cannot obtain *K*, which is only known to GWN. Thus, our authentication scheme ensures session key security.

• The proposed scheme provides an efficient password change phase:

In general, when a password change occurs, it is encouraged for the verification process to be carried out without any assistance from the GWN to ensure user friendliness and efficiency [24]. Our proposed scheme performs existing password checks in the self-verification process within the smart card. After checking the process through $B_{i}^{*}=B_{i}$, the computed values $\left(A_{i}^{new},B_{i}^{new}\right)$ from the new password $PW_{i}^{new}$ will be switched with the existed values $\left(A_{i},B_{i}\right)$ in a convenient and efficient way.

• The proposed scheme withstands gateway node bypass attacks:

During the authentication phase of our scheme, the attacker may try to construct the message $\langle DID_{i},M_{G,S_{j}},M_{j},T_{2}\rangle$ using the parameters $\left\{A_{i},B_{i},C_{i},D_{i},h\left(\cdot \right),H\left(\cdot \right)\right\}$ stored in the stolen smart card [31] in order to impersonate a legitimate GWN. However, the attacker cannot compute $X_{S_{j}}=h(SID_{j}\left||K\right)$ because *K* is not public information. Thus, the attacker cannot construct a sufficient message to cheat $S_{j}$. Eventually, the attacker cannot impersonate a valid GWN.

• The proposed scheme withstands off-line identity guessing attacks:

Suppose that the attacker extracts all of the secret information $\left\{A_{i},B_{i},C_{i},D_{i},h\left(\cdot \right),H\left(\cdot \right)\right\}$ from the smart card and intercepts $U_{i}$’s login request $\langle DID_{i},M_{U_{i},G},C_{i},T_{1}\rangle$. Using these values, the attacker may try to guess the correct identity $ID_{i}^{\prime }$ through $TID_{i}^{\prime }=h(ID_{i}^{\prime }\left||u\right)$, $HID_{i}^{\prime }=DID_{i}⊕TID_{i}^{\prime }$, $K^{\prime }=C_{i}⊕HID^{\prime }$, $HPW_{i}^{\prime }=HID_{i}^{\prime }⊕h(TID_{i}^{\prime }\left|\right|K^{\prime })$ and $B_{i}^{\prime }=h(DID_{i}⊕TID_{i}^{\prime }⊕h(TID_{i}^{\prime }\left|\right|K^{\prime })||DID_{i}⊕TID_{i}^{\prime })$. However, in order to successfully guess the $ID_{i}^{\prime }$, the attacker should know the random number *u*. Even though the attacker knows the $D_{i}$, the attacker fails to compute $u=D_{i}⊕H\left(Bio_{i}\right)$ because $H(Bio_{i})$ is not public information. Therefore, our proposed scheme can withstand an off-line identity guessing attack.

### 5.2. Authentication Proof Using BAN Logic

In this subsection, we use BAN logic to verify the legitimacy of the session keys distributed to participants who communicate in the proposed scheme. BAN logic [36] is applied as a well-known formal logic to analyze the security of cryptographic protocols. The basic notation for BAN logic is as follows.

- U◃C: *U* sees condition *C*.
- U∣≡C: Condition *C* is believed by *U*
- ♯(C): It makes a fresh *C*.
- U∣∼C: *U* expresses the condition *C*.
- $U\overset{K}{⟷}S$: *U* and *S* share a secret key *K*.
- U⇒C: Condition *C* is handled by *U*.
- ${\left(C\right)}_{K}$: Perform the hash operation on *C* using *K*.

BAN logic also offers five logic rules as follows.

- Rule 1. Message-meaning rule: $\frac{U|\equiv U\overset{K}{↔}S,U◃<C{>}_{K}}{U|\equiv S|∼C}$: if *U* trusts that the key *K* is shared with *S*, *U* sees the *C* combined with *K*, then *U* trusts *S* once said *C*.
- Rule 2. Nonce-verification rule: $\frac{U|\equiv \#(C),U|\equiv S|∼C}{U|\equiv S|\equiv C}$: if *U* trusts that *C*’s freshness and *U* trusts *S* once said *C*, then *U* trusts that *S* trusts *C*.
- Rule 3. Believe rule: $\frac{U|\equiv C,U|\equiv M}{A|\equiv (C,M)}$: if *U* trusts *C* and *M*, (C,M) are also trusted by *U*.
- Rule 4. Freshness-conjuncatenation rule: $\frac{U|\equiv \#(C)}{A|\equiv \#(C,M)}$: if freshness of *C* is trusted by *U*, then *U* can trust the freshness of full condition.
- Rule 5. Jurisdiction rule: $\frac{U|\equiv S|\Rightarrow C,U|\equiv S|\equiv C}{U|\equiv C}$: if *U* trusts that *S* has jurisdiction over *C*, and *U* trusts that *S* trusts a condition *C*, then *U* also trusts *C*.

Through our analysis, we will intend to satisfy the following four goals.

- Goal 1: $U_{i}|\equiv \left(U_{i}\overset{K_{S}}{⟷}S_{j}\right)$
- Goal 2: $S_{j}|\equiv \left(U_{i}\overset{K_{S}}{⟷}S_{j}\right)$
- Goal 3: $U_{i}|\equiv S_{j}|\equiv \left(U_{i}\overset{K_{S}}{⟷}S_{j}\right)$
- Goal 4: $S_{j}|\equiv U_{i}|\equiv \left(U_{i}\overset{K_{S}}{⟷}S_{j}\right)$

Next, all transmitted messages can be transmuted into an idealized form as follows.

- Message 1: $U_{i}\to GWN$ : ${\left(ID_{i},HPW_{i},K,T_{1}\right)}_{HID_{i}}$
- Message 2: $GWN\to S_{j}$ : ${\left(ID_{i},SID_{j},R,T_{2}\right)}_{X_{S_{j}}}$
- Message 3: $S_{j}\to GWN$ : ${\left(ID_{i},R,T_{3}\right)}_{X_{S_{j}}}$
- Message 4: $GWN\to U_{i}$ : ${\left(ID_{i},K,R,T_{4}\right)}_{HID_{i}}$

In order to analyze our authentication mechanism, we define some assumptions as follows.

- A1: $GWN|\equiv ♯(T_{1})$
- A2: $S_{j}|\equiv ♯\left(T_{2}\right)$
- A3: $GWN|\equiv ♯(T_{3})$
- A4: $U_{i}|\equiv ♯\left(T_{4}\right)$
- A5: $GWN|\equiv (GWN\overset{X_{S_{j}}}{⟷}S_{j})$
- A6: $S_{j}|\equiv \left(GWN\overset{X_{S_{j}}}{⟷}S_{j}\right)$
- A7: $U_{i}|\equiv \left(U_{i}\overset{HID_{i}}{⟷}GWN\right)$
- A8: $GWN|\equiv (U_{i}\overset{HID_{i}}{⟷}GWN)$
- A9: $U_{i}|\equiv S_{j}\Rightarrow \left(U_{i}\overset{K_{S}}{⟷}S_{j}\right)$
- A10: $S_{j}|\equiv U_{i}\Rightarrow \left(U_{i}\overset{K_{S}}{⟷}S_{j}\right)$

Now, we describe our main proof as follows. In order to describe our proof, we use predefined information, including five logic rules, four messages and ten assumptions.

- According to the Message 1, we could derive the following:
  V1: $GWN◃{\left(ID_{i},HPW_{i},K,T_{1}\right)}_{HID_{i}}$
- Based on Assumption A8 and Rule 1, we derive:
  V2: $GWN|\equiv U_{i}|∼{\left(ID_{i},HPW_{i},K,T_{1}\right)}_{HID_{i}}$
- Based on Assumption A1 and Rule 4, we derive:
  V3: $GWN|\equiv ♯{\left(ID_{i},HPW_{i},K,T_{1}\right)}_{HID_{i}}$
- Based on V2, V3 and Rule 2, we derive:
  V4: $GWN|\equiv U_{i}|\equiv {\left(ID_{i},HPW_{i},K,T_{1}\right)}_{HID_{i}}$
- According to Message 2, we derive:
  V5: $S_{j}◃{\left(ID_{i},SID_{j},R,T_{2}\right)}_{X_{S_{j}}}$
- Based on Assumption A6 and Rule 1, we derive:
  V6: $S_{j}|\equiv GWN|∼{\left(ID_{i},SID_{j},R,T_{2}\right)}_{X_{S_{j}}}$
- Based on Assumption A2 and Rule 4, we derive:
  V7: $S_{j}|\equiv ♯{\left(ID_{i},SID_{j},R,T_{2}\right)}_{X_{S_{j}}}$
- Based on V6, V7 and Rule 2, we derive:
  V8: $S_{j}|\equiv GWN|\equiv {\left(ID_{i},SID_{j},R,T_{2}\right)}_{X_{S_{j}}}$
- According to Message 3, we derive:
  V9: $GWN◃{\left(ID_{i},R,T_{3}\right)}_{X_{S_{j}}}$
- Based on Assumption A5 and Rule 1, we derive:
  V10: $GWN|\equiv S_{j}|∼{\left(ID_{i},R,T_{3}\right)}_{X_{S_{j}}}$
- Based on Assumption A3 and Rule 4, we derive:
  V11: $GWN|\equiv ♯{\left(ID_{i},R,T_{3}\right)}_{X_{S_{j}}}$
- Based on V10, S11 and Rule 2, we derive:
  V12: $GWN|\equiv S_{j}|\equiv {\left(ID_{i},R,T_{3}\right)}_{X_{S_{j}}}$
- According to Message 4, we derive:
  V13: $U_{i}◃{\left(ID_{i},K,R,T_{4}\right)}_{HID_{i}}$
- Based on Assumption A7 and Rule 1, we derive:
  V14: $U_{i}|\equiv GWN|∼{\left(ID_{i},K,R,T_{4}\right)}_{HID_{i}}$
- Based on Assumption A4 and Rule 4, we derive:
  V15: $U_{i}|\equiv ♯{\left(ID_{i},K,R,T_{4}\right)}_{HID_{i}}$
- Based on V14, V15 and Rule 2, we derive:
  V16: $U_{i}|\equiv GWN|\equiv {\left(ID_{i},K,R,T_{4}\right)}_{HID_{i}}$
- Based on V12, V16 and the session key $K_{S}=f\left(DID_{i},R\right)$, we derive:
  V17: $U_{i}|\equiv S_{j}|\equiv \left(U_{i}\overset{K_{S}}{⟷}S_{j}\right)$ (Goal 3)
- Based on V4, V8 and the session key $K_{S}=f\left(DID_{i},k_{i}⊕HPW_{i}⊕HID_{i}\right)$, we derive:
  V18: $S_{j}|\equiv U_{i}|\equiv \left(U_{i}\overset{K_{S}}{⟷}S_{j}\right)$ (Goal 4)
- Based on Assumption A9, V17 and Rule 5, we derive:
  V19: $U_{i}|\equiv \left(U_{i}\overset{K_{S}}{⟷}S_{j}\right)$ (Goal 1)
- Based on assumption A10, V18 and Rule 5, we derive:
  V20: $S_{j}|\equiv \left(U_{i}\overset{K_{S}}{⟷}S_{j}\right)$ (Goal 2)

The above description clearly shows that $U_{i}$, GWN and $S_{j}$ achieve the mutual authentication property. In addition, based on Goal 1, Goal 2, Goal 3 and Goal 4, we can assure that the session key $K_{S}$ is securely shared between them.

## 6. Formal Security Proof of the Proposed Scheme

In this section, we have demonstrated that the proposed scheme is secure through a formal proof using the random oracle model. First, we specify a cryptographic one-way hash function as follows.

**Definition** **1.** A hash function $f:{\left\{0,1\right\}}^{*}\to {\left\{0,1\right\}}^{n}$ is a one-direction function [38,39] that takes the input $x\in {\left\{0,1\right\}}^{*}$ of arbitrary length and outputs a bit string with a fixed-length $f\left(x\right)\in {\left\{0,1\right\}}^{n}$, which is referred to as the “message digest” or “hash value”. When using cryptographic hash functions, the following three common levels of security must be considered:

- It is impossible to acquire the input *x* under the conditions of the hash value y=h(x) and the given hash function h(·).
- It is impossible to acquire another input $x^{\prime }$, when given the input *x* and $f\left(x^{\prime }\right)=f\left(x\right)$.
- It is impossible to acquire the inputs $\left(x,x^{\prime }\right)$, where $x\neq x^{\prime }$, when given $f\left(x\right)=f\left(x^{\prime }\right)$.

Reveal: Given the hash result y=h(x), this random oracle will unconditionally output the input *x*.

**Theorem** **1.** *A one-way hash function h(·) is assumed to operate like an oracle. Under this assumption, our proposed mechanism is provably secure against an attacker A to protect $U_{i}$’s personal information, such as identity $ID_{i}$, password $PW_{i}$, biometrics $Bio_{i}$ and the GWN’s secret key K*.

**Proof.**  A similar method as that used in [26] is applied in our authentication mechanism to formally verify the security. For the proof, we assume that an attacker A is able to derive $U_{i}$’s identity $ID_{i}$, password $PW_{i}$, biometrics $Bio_{i}$ and the GWN’s secret key *K*. For this, A runs the experimental algorithm that is shown in Algorithm 1, $EXP1_{HASH,A}^{AUAKAS}$ for our anonymous user authentication with key agreement scheme (AUAKAS). We define the success probability for $EXP1_{HASH,A}^{AUAKAS}$ as $Success1_{HASH,A}^{ABUAKAS}=\left|Pr\left[EXP1_{HASH,A}^{ABUAKAS}=1\right]-1\right|$, where Pr(·) means the probability of $EXP1_{HASH,A}^{AUAKAS}$. The advantage function for this experiment becomes $Adv_{HASH,A}^{ABUAKAS}\left(t,q_{R}\right)=max_{A}\left\{Success1_{HASH,A}^{ABUAKAS}\right\}$ in which the maximum is determined by three factors: all of A, the execution time *t* and the number of queries $q_{R}$ derived from the Reveal oracle. If attacker A is assumed to be able to resolve the hash function problem, A could directly obtain $U_{i}$’s identity $ID_{i}$, password $PW_{i}$, biometrics $Bio_{i}$ and the GWN’s secret key *K*. Refer to the attack experiment described in Algorithm 1. In this case, A will discover the complete connections between $U_{i}$ and GWN. However, it is computationally infeasible to invert a one-way hash function h(·), i.e., $Adv_{HASH,A}^{AUAKAS}\left(t\right)\leq \epsilon$, ∀ϵ>0. Then, we have $Adv_{HASH,A}^{AUAKAS}\left(t,q_{R}\right)\leq \epsilon$, since $Adv_{HASH,A}^{AUAKAS}\left(t,q_{R}\right)$ depends on $Adv_{HASH,A}^{AUAKAS}\left(t\right)$. Therefore, our proposed scheme is provably secure against the attacker A for deriving $ID_{i},PW_{i},Bio_{i}$ and *K*. ☐
**Algorithm 1:** Algorithm $EXP1_{HASH,A}^{AUAKAS}$. 1. Eavesdrop login request message $\langle DID_{i},M_{U_{i},G},C_{i},T_{1}\rangle$ during the login phase. 2. Call the Reveal oracle. Let $\left(TID_{i}^{\prime },HPW_{i}^{\prime },HID_{i}^{\prime },T_{1}^{\prime }\right)\leftarrow Reveal\left(M_{U_{i},G}\right)$ 3. Call the Reveal oracle. Let $\left(ID_{i}^{\prime },u^{\prime }\right)\leftarrow Reveal\left(TID_{i}^{\prime }\right)$ 4. Computes $DID_{i}^{\prime }=h(ID_{i}^{\prime }\left|\right|u^{\prime })⊕HID_{i}^{\prime }$ 5. **if $\left(DID_{i}^{\prime }=DID_{i}\right)$ then** 6.    **Accepts $ID_{i}^{\prime }$ as the correct $ID_{i}$ of user $U_{i}$** 7.    **Call the Reveal oracle. Let $\left(PW_{i}^{\prime },Bio_{i}^{\prime }\right)\leftarrow Reveal\left(HPW_{i}^{\prime }\right)$** 8.    **Computes $M_{U_{i},G}^{\prime }=h(TID_{i}^{\prime }||h(PW_{i}^{\prime }||Bio_{i}^{\prime })||HID_{i}^{\prime }\left|\right|T_{1}^{\prime })$** 9.    **if $\left(M_{U_{i},G}^{\prime }=M_{U_{i},G}\right)$ then** 10.       **Accepts $Bio_{i}^{\prime }$ and $PW_{i}^{\prime }$ as the correct $Bio_{i}$ and $PW_{i}$ of user $U_{i}$** 11.       **Call the Reveal oracle. Let $\left(HID_{i}^{\prime \prime },K^{\prime }\right)\leftarrow Reveal\left(C_{i}\right)$** 12.       **if $\left(HID_{i}^{\prime \prime }=HID_{i}^{\prime }\right)$ then** 13.          **Accept $K^{\prime }$ as the correct secret key**
*K*
**of gateway node GWN** 14.          **return 1 (Success)** 15.       **else** 16.          **return 0** 17.       **end if** 18.    **else** 19.       **return 0** 20.    **end if** 21. **else** 22.    **return 0** 23. **end if**

**Theorem** **2.** The one-way hash function h(·) is assumed to perform as an oracle, and the smart card for $U_{i}$ is stolen by an adversary A. Under these assumptions, our proposed mechanism is secure against an adversary A to derive the password $PW_{i}$ of a user $U_{i}$.

**Proof.**  We assume that an attacker A is able to derive the $U_{i}$’s password $PW_{i}$ after extracting the parameters $\left\{A_{i},B_{i},C_{i},h\left(\cdot \right),H\left(\cdot \right)\right\}$ stored in the smart card by physically monitoring its power consumption [31]. A then runs the experimental algorithm $EXP2_{HASH,A}^{AUAKAS}$ that is shown in Algorithm 2. We define the success probability for $EXP2_{HASH,A}^{AUAKAS}$ as $Success2_{HASH,A}^{ABUAKAS}=\left|Pr\left[EXP2_{HASH,A}^{ABUAKAS}=1\right]-1\right|$, where Pr(·) means the probability of $EXP2_{HASH,A}^{AUAKAS}$. The advantage function for this experiment becomes $Adv2_{HASH,A}^{ABUAKAS}\left(t_{2},q_{R}\right)=max_{A}\left\{Success2_{HASH,A}^{ABUAKAS}\right\}$ in which the maximum is determined by three factors: all of A, the execution time $t_{2}$ and the number of queries $q_{R}$ derived from the Reveal oracle. If $Adv2_{HASH,A}^{AUAKAS}\left(t_{2}\right)\leq \epsilon$, ∀ϵ>0, our scheme is provably secure against the attacker A to derive $PW_{i}$. According to the attack experiment described in Algorithm 2, A could obtain $U_{i}$’s password $PW_{i}$ if A is able to resolve the hash function problem. However, as shown in Definition 1, it is computationally infeasible to invert a one-way hash function h(·). Then, we have $Adv2_{HASH,A}^{AUAKAS}\left(t_{2},q_{R}\right)\leq \epsilon$, since $Adv2_{HASH,A}^{AUAKAS}\left(t_{2},q_{R}\right)$ depends on $Adv2_{HASH,A}^{AUAKAS}\left(t_{2}\right)$. As a result, the proposed scheme is provably secure against attacker A to derive $PW_{i}$ even if the smart card is stolen by A. ☐
**Algorithm 2:** Algorithm $EXP2_{HASH,A}^{AUAKAS}$. 1. Extract the information $\left\{A_{i},B_{i},C_{i},D_{i},h\left(\cdot \right),H\left(\cdot \right)\right\}$ stored in the smart card      through physically monitoring its power consumption [31]. 2. Call the Reveal oracle. Let $\left(HPW_{i}^{\prime },TID_{i}^{\prime },HID_{i}^{\prime }\right)\leftarrow Reveal\left(A_{i}\right)$ 3. Call the Reveal oracle. Let $\left(PW_{i}^{\prime },Bio_{i}^{\prime }\right)\leftarrow Reveal\left(HPW_{i}^{\prime }\right)$ 4. Computes $HID_{i}^{\prime }=A_{i}⊕h(h(PW_{i}^{\prime }||Bio_{i}^{\prime })||TID_{i}^{\prime })$ 5. Computes $B_{i}^{\prime }=h(HPW_{i}^{\prime }||HID_{i}^{\prime })=h(h(PW_{i}^{\prime }||Bio_{i}^{\prime })||A_{i}⊕h(h(PW_{i}^{\prime }||Bio_{i}^{\prime })||TID_{i}^{\prime }))$ 6. **if $\left(B_{i}^{\prime }=B_{i}\right)$ then** 7.    **Accepts $PW_{i}^{\prime }$ as the correct $PW_{i}$ of user $U_{i}$** 8.    **return 1 (Success)** 9. **else** 10.    **return 0** 11. **end if**

## 7. Performance Analysis of the Proposed Scheme

In this section, we performed a comparison of the computational costs and execution time for the proposed scheme relative to other, related schemes [10,12,13,14,15]. In general, the computational cost is examined based on the respective operations in the authentication protocol. Accordingly, this analysis of the computational cost concentrates on the operations that are conducted by the participant, such as $U_{i}$, GWN and $S_{j}$. To evaluate the computational costs, we define the following computational parameters.

- $T_{H}$: the time to execute a one-way hash/pseudo-random function/biohash function.
- $T_{X}$: the time to execute a XOR operation.
- $T_{E}$: the time to execute a ECC multiplication.
- $T_{F}$: the time to execute a fuzzy extractor.

Table 3 provides a summary of the comparison of the computational overhead of the related schemes [10,12,13,14,15]. The results show that Khan and Alghathbar’s scheme [10], Vaidya et al.’s scheme [12], Kim et al.’s scheme [13], Change et al.’s scheme [14], Park and Park’s scheme [15] and the proposed scheme require total computational overheads of $16T_{H}+6T_{X}$, $30T_{H}+24T_{X}$, $37T_{H}+30T_{X}$, $37T_{H}+21T_{X}$, $39T_{H}+19T_{X}+3T_{F}+4T_{E}$ and $34T_{H}+15T_{X}$, respectively.

Based on the total cost results in Table 3, we have performed an experiment on the execution time to obtain an objective comparison between our scheme and other related schemes [10,12,13,14,15]. The following methods are generally used to measure the execution time for the authentication protocol: (i) determine computational overhead; (ii) measure the execution time of the cryptographic operations used in the protocol; and (iii) substitute the measured time obtained by (ii) into (i). We have measured the execution times using these measurement methods, and the results are shown in the execution time field of Table 3.

The results of the simulation in Li et al.’s and Wazid et al.’s research [40,41] show that the actual execution time for the cryptographic one-way hash function $T_{H}$ and ECC multiplication $T_{E}$ is 0.0005 s and 0.063 s, respectively. In addition, according to [41], the execution time of the fuzzy extractor operation $T_{F}$ is almost the same as the ECC multiplication operation $T_{E}$. Thus, we assumed that the time consumption of these two operations is the same. On the other hand, XOR operation $T_{X}$ is not considered in our measurement because the execution time of the XOR operation $T_{X}$ is extremely short. Based on the $T_{H}\approx 0.0005$, $T_{E}\approx 0.063$, $T_{F}\approx 0.063$ and the total computation cost, we finally analyze the execution time. As shown in Table 3, we observed that the execution time of our proposed scheme is of only 0.017 s ($34T_{H}$ ≈ 34 × 0.0005 s), so it can be considered as a negligible significance. In contrast, the execution times of Kim et al.’s scheme [13], Chang et al.’s scheme [14] and Park and Park’s scheme [15] are 0.0185 s ($37T_{H}$ ≈ 37 × 0.0005 s), 0.0185 s ($37T_{H}$ ≈ 37 × 0.0005 s) and 0.4605 s ($39T_{H}+3T_{F}+4T_{E}$ ≈ 39 × 0.0005 s + 7 × 0.063 s), respectively. Therefore, our scheme turned out to have a slightly better efficiency than these schemes [13,14,15]. Even if our scheme requires slightly more computation time than Khan and Alghathbar’s scheme [10] and Vaidya et al.’s scheme [12], this is acceptable because our scheme has more effective security features and a higher security level, as shown in Table 2.

## 8. Conclusions

In this paper, we have demonstrated that Chang et al.’s scheme has a number of critical weaknesses, and we propose an authentication mechanism with enhanced security to overcome these weaknesses. Our proposed scheme has been thoroughly verified in terms of its variety of security features, and the proof result demonstrates that our scheme can guarantee protection against various types of attacks, even if the smart card is stolen by an attacker. In addition, a performance comparison for the proposed scheme in relation to the schemes proposed in other studies was carried out, and we consider that our proposed scheme has sufficient efficiency for WSNs. 

## Figures and Tables

**Table 1:** Notations.

Value	Description
$U_{i}$	Remote user
$S_{j}$	Sensor node
GWN	Gateway node
$ID_{i}$, $PW_{i}$	Identity and password of $U_{i}$
$Bio_{i}$	Biometric information of $U_{i}$
$PW_{i}^{new}$	New password of $U_{i}$
*u*	Random number of $U_{i}$
$ID_{s}$	Identity of smart card
$TID_{i}$	Temporary identity for $U_{i}$’s next login
$SID_{j}$	Identity of $S_{j}$
*K*	Secret key generated by the GWN
$RN_{r},RN_{G},R$	Random numbers
h(·)	One-way hash function
H(·)	Biohash function
f(x,k)	Pseudo-random function of variable *s* with key *k*
X||Y	Concatenate operation
⊕	XOR operation
$T_{1}$, $T_{2}$, $T_{3}$, $T_{4}$	Current time stamp values
$K_{S}$	Session key
ΔT	The maximum of the transmission delay time

**Table 2:** Security comparison of our proposed scheme and other related schemes.

Features	Khan et al. [10]	Vaidya et al. [12]	Kim et al. [13]	Chang et al. [14]	Park et al. [15]	Our Scheme
User anonymity	×	×	√	√	√	√
Mutual authentication	×	√	√	√	√	√
Stolen smart card attack	×	×	×	×	√	√
Replay attack	√	√	√	√	√	√
Off-line PW guessing attack	×	√	√	×	√	√
$U_{i}$ impersonation attack	×	×	√	×	√	√
$S_{j}$ impersonation attack	×	√	×	√	√	√
Password verification	√	√	√	√	√	√
Session key verification	×	×	×	×	×	√
Privileged-insider attack	√	√	√	√	√	√
Session key security	×	×	×	×	√	√
Efficient password change	√	√	√	√	√	√
GWN bypass attack	×	×	√	√	√	√
Off-line ID guessing attack	×	×	√	√	√	√
No verifier table	√	√	√	×	×	√
Formal proof	×	√	×	√	√	√

**Table 3:** Comparison of the computational cost between our scheme and other hash-based schemes.

Phases		Khan et al. [10]	Vaidya et al. [12]	Kim et al. [13]	Chang et al. [14]	Park et al. [15]	Proposed Scheme
Registration	$U_{i}$	$1T_{H}$	$1T_{H}$	$2T_{H}+1T_{X}$	$2T_{H}+1T_{X}$	$1T_{H}+1T_{F}$	$3T_{H}$
	GWN	$2T_{H}+1T_{X}$	$4T_{H}+2T_{X}$	$6T_{H}+3T_{X}$	$5T_{H}+3T_{X}$	$5T_{H}+3T_{X}$	$3T_{H}+3T_{X}$
	$S_{j}$	−	−	−	−	−	−
Login	$U_{i}$	$3T_{H}+1T_{X}$	$6T_{H}+4T_{X}$	$7T_{H}+5T_{X}$	$7T_{H}+4T_{X}$	$6T_{H}+3T_{X}+1T_{F}+1T_{E}$	$6T_{H}+2T_{X}$
	GWN	−	−	−	−	−	−
	$S_{j}$	−	−	−	−	−	−
Authen tication	$U_{i}$	−	$2T_{H}+3T_{X}$	$2T_{H}+4T_{X}$	$4T_{H}+2T_{X}$	$4T_{H}+2T_{X}+1T_{E}$	$2T_{H}+2T_{X}$
	GWN	$5T_{H}+2T_{X}$	$6T_{H}+6T_{X}$	$8T_{H}+8T_{X}$	$6T_{H}+4T_{X}$	$11T_{H}+4T_{X}$	$8T_{H}+5T_{X}$
	$S_{j}$	$2T_{H}$	$3T_{H}+2T_{X}$	$3T_{H}+2T_{X}$	$4T_{H}+1T_{X}$	$4T_{H}+1T_{X}+2T_{E}$	$4T_{H}+1T_{X}$
Password change	$U_{i}$	$3T_{H}+2T_{X}$	$8T_{H}+6T_{X}$	$9T_{H}+7T_{X}$	$9T_{H}+6T_{X}$	$8T_{H}+6T_{X}+1T_{F}$	$8T_{H}+2T_{X}$
	GWN	−	−	−	−	−	−
	$S_{j}$	−	−	−	−	−	−
Total cost		$16T_{H}+6T_{X}$	$30T_{H}+24T_{X}$	$37T_{H}+30T_{X}$	$37T_{H}+21T_{X}$	$39T_{H}+19T_{X}+3T_{F}+4T_{E}$	$34T_{H}+15T_{X}$
Execution time		≈0.008 s	≈0.015 s	≈0.0185 s	≈0.0185 s	≈0.4605 s	≈0.017 s
